# “Our daily life was mainly comprised of eating and sitting:” a qualitative analysis of adolescents’ experiences of inpatient eating disorder treatment in China

**DOI:** 10.1186/s40337-019-0250-6

**Published:** 2019-06-17

**Authors:** Yi Wu, Amy Harrison

**Affiliations:** Department of Psychology and Human Development, Institute of Education, University College London, 25 Woburn Square, London, WC1H 0AA UK

**Keywords:** Anorexia nervosa, Eating disorders, China, Inpatient treatment, Patient experience

## Abstract

**Background:**

Some individuals with Eating Disorders (EDs) require hospitalization to stabilize and improve life-threatening physical complications. The experiences of those receiving inpatient treatment for EDs are well-documented in western samples, but less is known about the experiences of those in China, the world’s most populous country in which the incidence of EDs may be increasing.

**Methods:**

This qualitative study aimed to use Interpretative Phenomenological Analysis to understand the experiences of four adolescents receiving inpatient treatment for EDs in China. Individual, semi-structured interviews were conducted.

**Results:**

Four themes emerged from the data: perceptions of the treatment received, peer influences during admission, the impact of treatment on wellbeing and participants’ sense of self.

**Conclusions:**

This is the first published study on the experience of Chinese participants with EDs. Their experiences share commonalities with those reported by participants in Western studies and also illuminate differences in how EDs are understood and managed in inpatient settings in China. Cross-cultural collaborations will be important to share knowledge and practice.

## Plain English summary

Eating disorders are being reported more frequently in China, and while past research has largely explored the treatment experiences of those in Europe, the USA and Australia, little is known about Chinese participants’ experiences of treatment for an eating disorder. In this study, four adolescents receiving inpatient treatment for an eating disorder were interviewed and the experiences they shared are illustrated through four ‘themes’ which represent participants’ perceptions of the treatment they received, how their peers impacted them during their hospital admission, the degree to which treatment impacted their own wellbeing and the impact of treatment of their sense of self. Knowing more about how treatment is delivered in different countries will enable practitioners to collaborate around good practice for treating eating disorders.

## Background

Eating disorders (EDs), including anorexia nervosa (AN) and bulimia nervosa (BN) are psychiatric disorders with severe physical complications. Their typical onset is during adolescence and EDs have the highest death rate of any psychiatric illness [1]. Behaviors include food restriction, bingeing, self-induced vomiting and excessive exercise [2]. Life-time risk is increasing [3] and in the United Kingdom (UK), adolescents with EDs occupy the largest number of child and adolescent psychiatric beds [4]. Due to effects of starvation on the body, including cardiovascular risks, many people require hospitalization, which for the 20% who have the more severe and enduring form of illness, can be extensive and repeated [5].

Previous work has investigated the lived experiences of this group of individuals requiring hospitalization using qualitative methodologies. Studies from the UK [6–13], The Netherlands [14], Norway [15] and Australia [16] all report themes of control versus collaboration, removal from normality, the negative and positive impacts of peer relationships, transition and recovery, disconnection from the outside world, the role of meals and eating, battling the ED and taking responsibility. In these countries, inpatient treatment tends to involve physical rehabilitation alongside psychotherapies including cognitive behavioral therapy (CBT) and family therapy [17] and individuals may be admitted voluntary or detained and treated using legal frameworks like the UK’s Mental Health Act [18]. One key limitation of the current literature is that research on the experience of inpatient treatment is restricted to these western countries, perhaps because erroneously, it was once thought that EDs were culturally bound [19]. Therefore, little is known about experiences of inpatient treatment for EDs in countries such as China, which, hosting the world’s largest population, has a developing literature on the prominence of eating difficulties [20–27]. Indeed, although under-researched relative to western countries, EDs are becoming formidable public health challenges in China [28], showing significant increases in prevalence, with similar rates to the west [28–30], with which China shares thinness as a desirable trait [31]. Therefore, this study aimed to use Interpretive Phenomenological Analysis (IPA; [32]) to understand the experiences of four adolescents receiving inpatient treatment for EDs in mainland China.

## Methods

### Design

This qualitative inquiry employed a within-participants design.

### Participants

A purposive, volunteer sample of four Chinese adolescent girls (aged 16–19) admitted to a university hospital in Beijing for treatment of anorexia nervosa (AN) was recruited to take part in the study. The study was publicized through an online forum for families affected by EDs in the region. The researcher was given permission by the forum moderator to advertise the study in this space. Participants aged 16 to 20 of any gender were eligible to take part if they were receiving inpatient treatment for an ED and could consent to agree to speak about their experiences. Participants’ identities are protected through the use of pseudonyms.

### Measures

A semi-structured interview was selected due to its flexibility [33] and ability to permit unforeseen ideas to emerge [34] and was developed and piloted beforehand. The interview schedule consisted of a series of open-ended questions informed via a systematic review of the literature on the inpatient experience, discussions with service users and experts in the field. The semi-structured interview is provided in Appendix.

### Procedure

Participants were provided with an information sheet, and, having consented to participation, were interviewed individually, using video conferencing and the interview was audio recorded. Participants were informed that they had the right to withdraw at any point with no impact on their hospital treatment. Interviews lasted around 30 min. At the end, participants received a full debrief which involved discussing the project in more depth with participants, answering any outstanding questions that participants might have and providing a space to discuss and provide support around any issues or challenges that had arisen for them during the interviews. Interviews were conducted in Chinese, transcribed verbatim and then professionally translated into English. A sample back translation was conducted by an expert native speaker to ensure accuracy of meaning. The study received ethical approval from the University College London Research Ethics Committee (Ref 17.406) and was conducted in keeping with the principles of the Declaration of Helsinki and in agreement with the hospital where the participants were being treated.

### Data analysis

An inductive approach [35] was used to permit the generation of themes from the data. Interpretative Phenomenological Analysis (IPA; [32, 36]), an ideographic approach used widely in health and clinical psychology research (e.g. [37]) and in similar studies exploring the experience of inpatient treatment for EDs [6] was employed. IPA was selected due to its focus on understanding in-depth psychological perspectives and lived experience in small samples [36]. IPA takes both phenomenology and social constructionism into consideration, sitting between the critical realist and social constructionist perspectives [38]. IPA assumes all individuals are self-interpretative and underlines how individuals reflect upon and make sense of their experiences [39] and is associated with the hermeneutic (i.e. to interpret) tradition [40], adopting a ‘double hermeneutic,’ such that researchers are required to interpret the participants’ interpretations of their experience while being affected by their preoccupied experience and/or understanding [32]. An analytic process involving three steps [41] was followed. First, interviews were analyzed individually to permit immersion in the detail of the data. Meanwhile, all audio-recordings and transcripts were revised several times, and the intonation and expression were carefully noted down to increase precision and clarity. This was particularly important given that interviews were conducted in Chinese and then translated into English. Where direct translation was not possible, comments were provided to aid the understanding of the second author, a non-Chinese speaker. Second, emergent themes were identified and clustered as per Smith et al. [41] which involved abstraction (naming the clusters of inter-correlated emergent themes), subsumption (making specific emergent themes which can fully represent other related themes as super-ordinate themes), polarization (detecting oppositional ideals), contextualization (recognizing contextual/narrative components within analyses), numeration (counting how frequent a theme was supported), and function (examining themes’ functions). Third, master themes, which represented shared superior patterns, were developed by bringing all identified themes from individual cases into collective consideration. This process was conducted independently by two coders (YW and AH) who met regularly to discuss and agree on the emerging and final thematic map.

## Results

The final sample consisted of four female participants all diagnosed on admission by a Consultant Psychiatrist with the binge-purge subtype of AN according to the DSM 5 [2]. They were aged 16 to 19. For three participants (75%), this was their second admission and for one participant (25%), this was their first admission. Participants had been unwell for a mean of 3.7 years (SD = 2.2). Four master themes emerged from the data: participants’ understanding of the nature of the treatment received, peer influences during the admission, the impact of treatment on wellbeing and participants’ sense of self. The section below explores each master theme and its subordinate themes in turn, using quotations taken from the transcripts to explain the themes/subthemes further.

### Master Theme 1: *Nature of the Treatment Received*

**Fig. 1:**
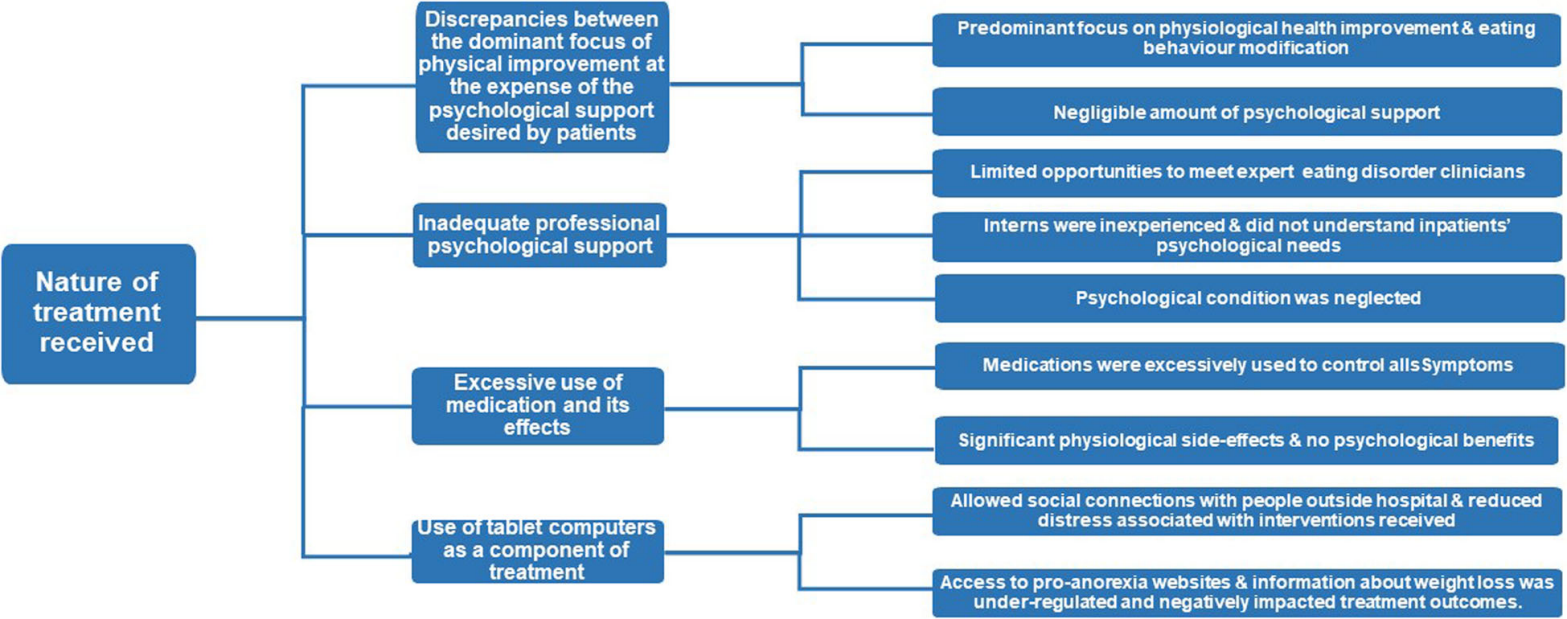
The master theme the “*Nature of treament received*” is presented in the furthest left-hand box, and subordinate themes are presented in the middle boxes. Supplementary explanations are provided in the furthest right-hand boxes provide further explanation. The connecting lines indicate the hierarchical organisation of the themes

The first master theme reflected participants’ ideas about the nature of the treatment received (Fig. 1).

#### Subordinate Theme 1: *Discrepancies between the dominant focus of physical improvement at the expense of the psychological support desired by participants*

The first subordinate theme reflects the discrepancy participants experienced between what they felt was the focus of the intervention they received and what they felt they needed. Participants all perceived that inpatient treatment mainly focused on modifying their eating behaviors and improving their physiological condition (i.e. nutrition improvement and weight gain) and that negligible attention was allocated to psychological improvement, which they felt was equally, if not more, important:

*“I felt they could have tried to provide more psychological interventions… I did not think they provided that much psychological intervention…”. (Bik)*

*“I felt that the nutrition intervention was the dominant intervention, and psychological intervention was the sub-intervention”. (Daiyu)*

*“Psychological help they provided was too little. They could not help soothe you psychologically or reduce your anxiety…they normally just asked about whether you felt any physical discomfort these days…they would prescribe medications to you…and some issues like changing your meal plan. And something like bathing or other very tiny little things. Psychological help was extremely rare”. (Ah Lam)*

Chun summarized her daily life as follows:

*“Our daily life was mainly composed of eating and sitting…”. (Chun)*

This combination of a lack of psychological care alongside weight restoration was experienced as problematic and traumatic by participants.

#### Subordinate Theme 2: *Inadequate professional psychological support*

The subordinate theme of there being inadequate psychological intervention from qualified professionals and little contact with the ED expert in the team further highlighted the contrast they experienced between what they reported to be an absence of psychological treatment alongside the treatment for the physiological complications of their illness:

*“During the whole period of receiving inpatient care, I only saw her (the ED expert team member) twice or three times, maximum. Once you started to live inside, she might talk to you once. And after a while, let’s say a month later, she would talk to you for the second time. And just before discharging you, she would talk to you again”. (Ah Lam)*

*“The chief doctor…actually I did not have that many chances to talk to her”. (Bik)*

Participants reported being matched with junior doctors, who were directly responsible for them and met people more frequently. However, participants perceived these doctors as inexperienced and felt they struggled to meet their psychological needs:

*“The doctors were quite perfunctory…these doctors were mainly interns and some of them were really being silly—they always misunderstood what you were talking about…they might be not that experienced and probably answered your questions with something irrelevant”. (Daiyu)*

#### Subordinate Theme 3: *Excessive use of medication and its effects*

A further subordinate theme reflected participants’ experiences of their illness being primarily treated using medications. They expressed concerns regarding over-use of medication:

*“If you cannot not sleep well, they give you sleeping pills…if you want to eat (binge), they would give you pills to suppress appetite. And if you want to exercise, they give you pills to sedate you. Everything in the ward is solved by taking medication”. (Ah Lam)*

Participants reported negative physiological outcomes from medication (e.g. over-reliance on medication, tolerance and withdrawal) and perceived that this treatment offered little psychological benefits:

*“They prescribed Lorazepam to control my anxiety…while I was taking it, it was not effective at all. But when I stopped taking it, I could not bear my condition…, my anxiety was extreme, I could not sit still and felt anxious all the time. And finally…I just re-took it and increased the dose”. (Ah Lam)*

*“My body became reliant on these medications... [when I stopped taking Olanzapine] I could not fall asleep and became very anxious”. (Chun)*

#### Subordinate Theme 4: *Use of tablet computers as a component of treatment*

The final subordinate theme was the use of tablet computers on the ward which participants were encouraged to use as part of treatment. For some, being permitted to use tablet computers was viewed as a positive component of treatment, as it lessened boredom and facilitated connections with others which distracted participants from their distress:

*“There were definitely more positive influences such as using the iPad which could help reduce anxiety and you could facetime your parents”. (Ah Lam)*

However, participants also noted that unmonitored access to tablet computers seemed at odds with the overall treatment approach, as they had access to nutritional and weight-loss information:

*“Others used their iPad to calculate the calories in their meals. I saw others going online to look at diets so that they could lose weight after discharge”. (Chun)*

What seemed to be implicit in these narratives was that participants perceived their psychological distress not to matter in the treatment approach, as they saw it to focus mainly on nutritional restoration. They desired psychological support to ‘soothe’ the distress related to re-feeding and wanted a relationship with staff members where they were taken seriously by those treating them which may point to a need for staff training and support around working with this patient group who have life-threatening forms of ED requiring inpatient treatment which will evoke anxiety in the staff members trying to help.

### Master Theme 2: Peer Influence

**Fig. 2:**
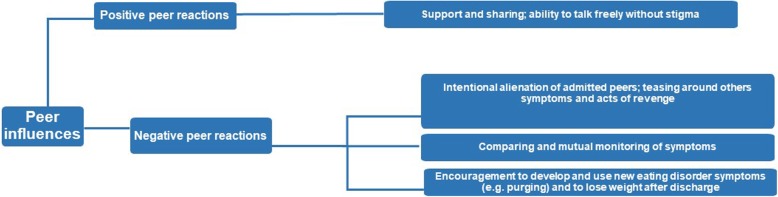
The master theme “*Peer Influence*” is presented in the furthest left-hand box, and subordinate themes are presented in the middle boxes. Supplementary explanations are provided in the furthest right-hand boxes provide further explanations. The connecting lines indicate the hierarchical organisation of the themes

The second master theme reflected participants’ experiences of peers during admission and reflected group dynamics on the ward (Fig. 2).

For all participants, the experience of being treated alongside peers with the same illness created a ‘bubble’ which they existed in during hospitalization:

*“Being with other girls inside [the hospital] was like living in a micro-society”. (Daiyu)*

Like many young people in English speaking countries, being on the ward was the participants’ first time living apart from their families. The group dynamics that seemed to involved elements of cooperation and conflict, with participants reporting on both positive and negative aspects of social interactions with their peers. Both authors were curious about whether some of these dynamics might reflect what it might be like for young people who were likely to be only children (due to the one child policy in China) to live with others of a similar age/generation perhaps for the first time.

#### Subordinate Theme 1: *Positive Peer Influences*

Participants discussed how being amongst peers with a similar illness provided a platform to freely talk about EDs without worrying about being stigmatized:

*“I had been keeping this secret (my ED) for an extremely long time without finding somebody to talk to. I think I have found someone to talk to here”. (Daiyu)*

The presence of peers also prevented some ED behaviors, as concerned peers reported concerning behaviors to the nurses:

*“And about vomiting… lots of people would definitely go to the nurses and report this issue. This stopped me from vomiting so much”. (Ah Lam)*

#### Subordinate Theme 2: *Negative Peer Influences*

There were also a number of negative peer influences, including a shared focus on food and weight:

*“We spent a significant amount of time together talking about weight gain and calorie intake and the like. I felt this did impact me more or less since. I felt my body had gained lots of weight and ...focusing on talking about weight, I felt psychologically uncomfortable and planned to lose weight”. (Bik)*

Participants discussed making comparisons with each other and reported spending significant amounts of time comparing food intake and who gained weight faster (or slower). This seemed to intensify fears around nutritional intake and weight gain:

*“I feel I was living in a space full of comparison, everything would be compared between us. They (peers) compared things like whose was larger or smaller, who ate more or less, who gained weight faster or slower”. (Chun)*

Peers negatively impacted one another through discussions around ED behaviors and weight:

*“Patients were discussing how to not gain weight and saying how anxious they were”. (Ah Lam)*

*“I felt there were more negative influences since I cared so much about the weight and they (peers) talked too much about the issues surrounding weight”. (Bik)*

“*I will be sometimes negatively influenced by them (those who don't want to recover)”. (Daiyu)*

Participants also discussed how to cope with weight gain in hospital by encouraging weight loss after discharge:

*“Being told (be peers) to lose weight after getting discharged can reduce that person’s anxiety a lot”. (Ah Lam)*

Participants reported learning new ED behaviors while in hospital and being encouraged to use them by other experienced people:

*“Some people would tell the others how they binged outside and taught the others who did not know how to purge before where to suppress and to release the pressure”. (Ah Lam)*

*“She taught me so many different ways to manually induce vomiting”. (Chun)*

Participants discussed bullying, teasing and alienation perpetrated by peers on the ward:

*“Some of them would intentionally create alienation between patients”.*

*“I realized that there was nobody who did not say bad things about the others (including care workers, nurses, patients and doctors)... And this made me feel living inside was extremely painful. I understood that I had negatively influenced the others (because of my physical symptoms), which had made me feel guilty already; they were still talking like that (telling each other I was disgusting), which made me even more stressed”. (Ah Lam)*

Sometimes these negative peer interactions resulted in acts of revenge from other experiencing people:

*“She hid half of the steam bun in her clothes… we (myself and another patient) decided to report this to the nurses. … just before she left (the hospital), she taunted me while the other patients were all there”. (Chun)*

These experiences seemed indicative of difficulties managing group dynamics and peer abuse on the ward which then allowed for new ED behaviors to be discussed and acquired, and for comparisons to be made amongst the group which may have maintained the ED or contributed to possible relapse on discharge.

### Master Theme 3: *Impact of Treatment on Wellbeing*

**Fig. 3:**
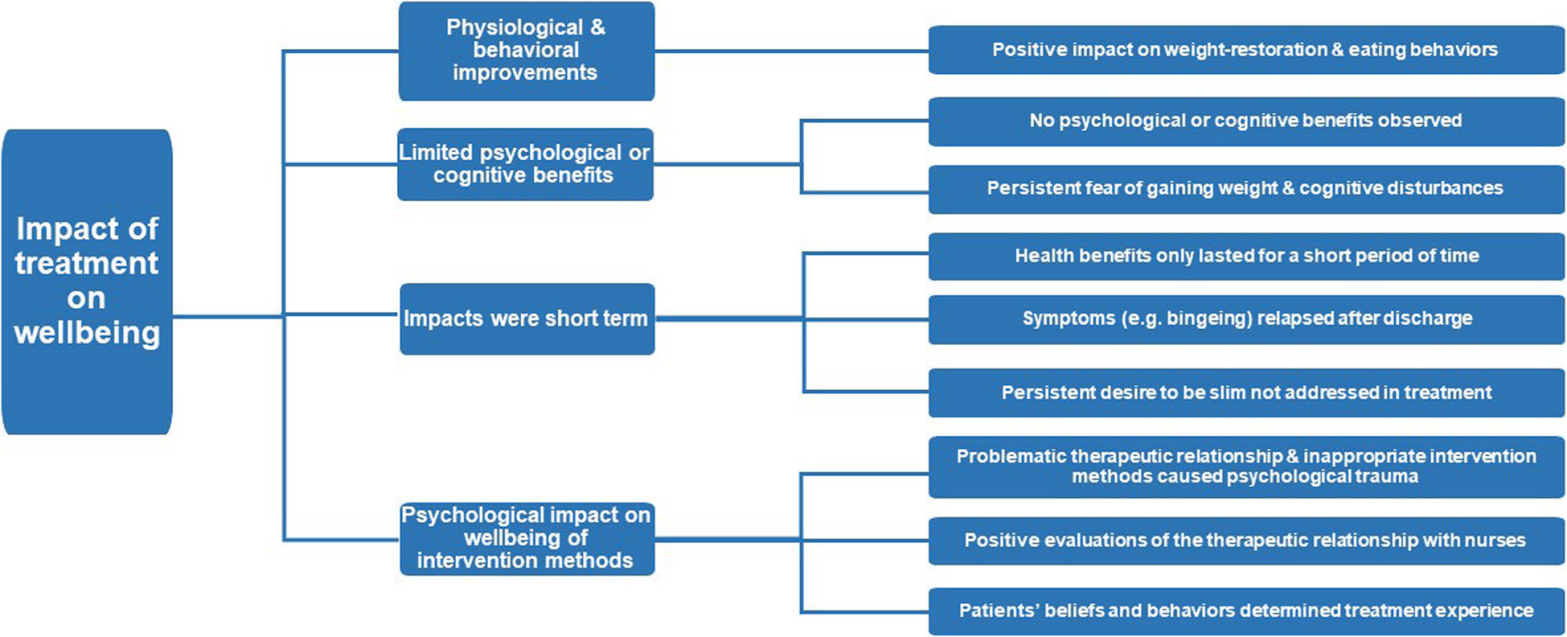
The master theme– *The Impact of Treatment on Wellbeing*–is presented in the furthest left-hand box, and subordinate themes are presented in the middle boxes. Supplementary explanations are provided in the furthest right-hand boxes provide further explanations. The connecting lines indicate the hierarchical organisation of the themes

The third master theme reflects an analysis of participant experiences related to the impacts of treatment on their wellbeing. This differs to the first master theme, in that rather than reporting participants’ perceptions of the treatment they received, this theme relates to what participants believed were the benefits of inpatient treatment for their ED (Fig. 3).

#### Subordinate Theme 1: *Physiological and behavioral improvements*

The first subordinate theme related to the physiological and behavioral improvements participants reported, like improvements in physical wellbeing (weight restoration) and a reduction in disruptive behaviors (bingeing and purging). Some participants discussed how inpatient treatment had saved their life:

*“It has already saved my life more than once or twice. Probably without receiving inpatient care I would have already died”. (Ah Lam)*

*“I have gained lots of weight during the period of receiving inpatient care…”. (Bik)*

*“Now I am eating normally and can stop bingeing and purging…. The only positive impact was on physical health, and the other impacts were all negative”. (Ah Lam)*

#### Subordinate Theme 2: *Limited psychological or cognitive benefits*

The second subordinate theme reflects a lack of improvement in psychological or cognitive functioning. Their experiences highlight that in spite of the physical improvement achieved through their hospitalization, they continued to experience heightened fear of gaining weight and other ED cognitions:

*“I did not find that the staff helped me with my anxieties about my weight… I was not helped psychologically, it was all about the physical improvements”. (Chun)*

#### Subordinate Theme 3: *Short-term impacts of treatment*

The third subordinate theme reflects frustrations with the longevity of behavioral improvements. Participants reported that while positive health outcomes were achieved, these were short-lived; meanwhile, more adverse long-term health outcomes were reported:

*“I think it did help in terms of short-term outcomes…but I did not see any long-term effects. After I got discharged from the hospital, the bingeing got even worse”. (Bik)*

*“These influences (the positive outcomes) only lasted for a short period of time”. (Daiyu)*

Participants said that behavioral improvements did not continue into the longer term. They felt that this was because their psychological wellbeing had been neglected:

*“I think it [bingeing and purging becoming even worse after discharge] might be because I have gained lots of weight during the period of receiving inpatient care, but I could not psychologically accept it…thus…I started to fast badly, and after a while, my bingeing emerged and my urge to eat got even stronger”. (Bik)*

Further, participants expressed ideas that longer-term change may not have been achieved because their over-valuation of slimness wasn’t addressed in treatment:

*“I still want to lose weight and keep slim. So I will not change my behaviors”. (Daiyu)*

#### Subordinate Theme 4: *Psychological impact of intervention methods on wellbeing*

The fourth subordinate theme reflected negative experiences of inpatient treatment which had a psychological impact. Participants perceived their inpatient treatment experience as traumatic and perhaps shameful:

*“The hospital is called mental health center, but it is just an asylum. So living inside can definitely induce psychological distress and discomfort”. (Ah Lam)*

*“The experience still brings me some psychological traumas. Sometimes, I dream about the events that happened during inpatient care. Anyway, they were nightmares”. (Bik)*

One aspects of participants’ negative experiences of treatment was a challenging therapeutic relationship between patients and nurses. For example, two participants reporting a damaging relationship with traumatic experiences and frightening memories:

*“Whenever they (the nurses who were extremely fierce) would be on duty tomorrow I could not sleep well in the night before, because of fear”. (Bik)*

*“The way the nurses treated us was not respectful at all…the nurses yelled at us…they did not treat you with dignity”. (Ah Lam)*

*“The most unacceptable intervention method for me was to be tied-up. The patients…every time while I saw this (others being ‘tied-up’ – mechanical restraint), I felt so sad…those being tied up would scream and cry their lungs out”. (Ah Lam)*

Other participants experienced the patient-nurse relationship as less traumatic and dangerous, and held contrary views, suggesting that this strong approach was needed. These participants explained that they dealt with the patient-nurse relationship through being extremely cooperative and obedient and reflected that this provoked positive interactions between themselves and the nurses:

*“At least they (nurses and care-workers) did this (being harsh, forcing us to eat, ‘protecting’ us – (here the participants is referring to the use of mechanical restraint)) for our health, and they were just following the doctors’ instructions…”. (Chun)*

*“It was possibly because I was being really cooperative, the nurses treated me well”. (Daiyu)*

### Master Theme 4: *Sense of self*

**Fig. 4:**
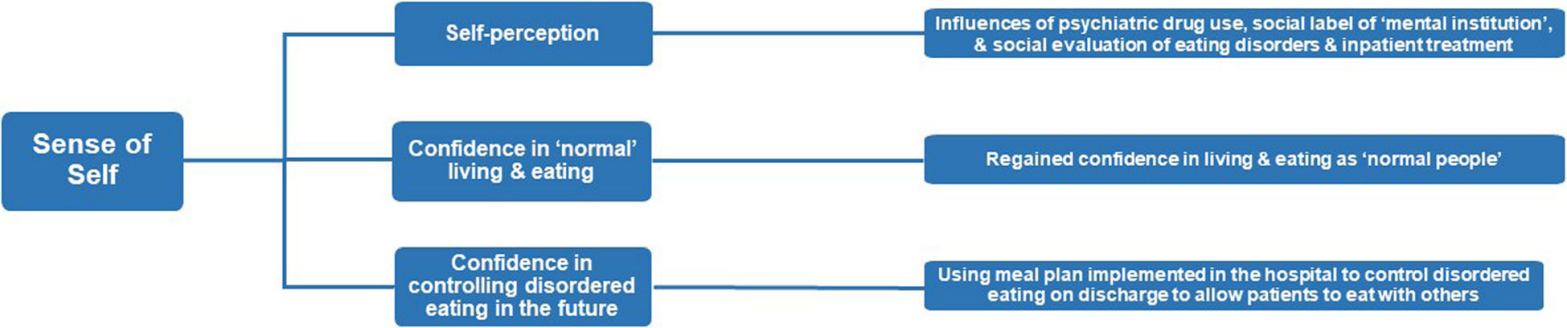
The master theme– Participants' *Sense of Self*– is presented in the furthest left-hand box, and subordinate themes are presented in the middle boxes. Supplementary explanations are provided in the furthest right-hand boxes provide further explanations. The connecting lines indicate the hierarchical organisation of the themes

This master theme related to how inpatient treatment had influenced participants’ sense of self (Fig. 4).

#### Subordinate theme 1: *Self-perception*

The first subordinate theme, self-perception, relates to participants’ self-evaluation having undergone inpatient treatment. This appeared to be strongly dominated by their experiences of being ‘psychiatric patients.’ Some participants reported negative self-evaluations:

*“I have been taking too many psychiatric drugs and this has made me think I am a psychopath”. (Ah Lam)*

However, other participants felt they needed medicine because they were ill and that this fact shouldn’t impact their self-evaluation:

*“I don't think taking medication means I am psychopathic.… I feel taking medication is normal while being ill”. (Daiyu)*

Nonetheless, all participants worried that this new experience which had contributed to their self-perception in some way might be perceived negatively by others:

*“Whenever I hear somebody else mentioning words like ‘mental institution’ I become very sensitive… And I cannot stop worrying about whether others will know I had an experience of being hospitalized”. (Ah Lam)*

This comment suggests that Ah Lam’s experience of their identity following the inpatient admission reflects more than an experience of being a ‘psychiatric patient’ and relates to the process of recruitment into the identity of a ‘disordered person.’ This highlights the power imbalance within the context of a setting like an inpatient unit.

*“I am worrying about how teachers and peers will evaluate me”. (Chun)*

These statements may also reflect the sense of shame and stigma that many participants felt around their diagnosis and the treatment their symptoms had required.

#### Subordinate Theme 2: *Confidence in ‘normal’ living and eating*

The second subordinate theme reflects how inpatient treatment had led to experiencing people incorporating a sense of normal versus abnormal eating behavior into their sense of self. They discussed their renewed confidence to be able to eat like others in their communities with the help of their meal plan:

*“I can generally understand the amount of food normal people consume every day”. (Bik)*

*“I am determined to eat meals normally by following the guidance set up by the hospital...while I was unable to control my disordered eating before, I can use this as a way to find a new balance and be more like normal people around food”. (Daiyu)*

*“Though ...I ate a lot and felt uncomfortable [during the admission], I was eating like a normal person. You would enjoy the food there [in hospital] and realized that you hadn't been eating normal meals for years”. (Daiyu)*

There was a sense that through treatment participants had become more able to ‘eat normally’ alongside others in their society:

*“I can live like a normal person”. (Chun)*

#### Subordinate Theme 3: *Confidence in controlling disordered eating in the future*

Finally, participants discussed how they planned to use what they had learnt in the hospital to manage their symptoms in the community in future:

*“I feel much more in charge of my eating behaviors and I think I can control my binges”. (Chun)*

*“My meal plan gives me confidence that I can continue to get better outside of the hospital”. (Bik)*

## Discussion

The aim of this study was to use Interpretive Phenomenological Analysis [32] to understand the experiences of four adolescents receiving inpatient treatment for EDs in mainland China. Four master themes emerged from the data: the first reflecting what participants perceived to be the nature of the treatment they received as inpatient; the second reflecting participants’ ideas around the positive and negative impacts of peers during their inpatient admission; the third relating to the experienced impacts of treatment on wellbeing and the fourth relating to the impact of inpatient treatment on participants’ sense of self.

The overall experience of these participants was mixed. They perceived inpatient treatment to offer and deliver welcome, positive, short-term improvements in their physical wellbeing, largely achieved through medication (infrequent) interactions with ED specialists and the presence of (some) supportive and understanding peers. For some, a strict, controlled ward environment was warranted as a means of saving lives. At the end of treatment, they felt like they could live and eat more ‘normally’ alongside others in their community. Conversely, participants felt that a combination of factors resulted in their psychological needs not being well met. These included the inexperience of the ‘intern doctors’ with whom they had more regular contact, ‘strict, cold, punitive’ nurses who often resorted to mechanical restraint, difficult interactions with (some) peers who encouraged competition and ED behaviors, over-reliance on medication and under-use of psychological therapies (which participants said they wanted). This seemed to reflect difficulties in understanding and managing difficult ED behaviors and the anxiety of the staff in working with this group of individuals. Because treatment benefits were largely limited to the restoration of physical health at any cost, credited with saving participants’ lives, they felt they were vulnerable to relapse after discharge. A clear message from these experiencing people was the need for a more psychologically driven treatment as a means of supporting longer-term recovery. Finding balance between physical interventions and psychological treatments is a discussion held in treatment settings in other countries like the UK [17]. Participants’ experiences of mechanical and chemical restraint may reflect the greater issue of the need for alternative ways of engaging the person in the process of change within an inpatient context. This highlights the need for improved training and support for staff in the uptake of interventions that support rather than potentially traumatize the experiencing person. Furthermore, this raises the question of fiscal support in the training and supervision of staff in the treatment of EDs, including in inpatient contexts. Adequate financial support for ED treatment is a challenge shared with many countries. It could be that these negative experiences could be improved through supporting staff with training so that they could have alternative, less-traumatic ways of supporting individuals.

Like Smith et al.’s [16], Coltan and Pistrang’s [6] and Offord et al.’s [10] UK studies, the positive impact of sharing experiences with understanding peers was similarly important to the UK and Chinese participants. The theme reflecting the contribution of the inpatient experience to participants’ sense of self in this study is corroborated by Smith et al.’s master theme reflecting the sense of self-discovery obtained through the inpatient experience. An interesting parallel between this study and Smith et al.’s [16] was that while both samples discussed the importance of supportive staff relationships; in this sample there were mixed experiences of what this actually looked like in practice, with some participants experiencing staff as cold, punitive and overly strict which they felt was unhelpful. Interpersonal relationships in treatment were also highlighted as being salient to participants in other UK studies such as Fox and Diab [7]. It was interesting to note the bullying and alienation reported by the Chinese participants. These strong negative narratives provoked an interesting discussion between the two authors (one from the UK and the other Chinese) about cultural differences regarding the expression of negative emotions and attitudes which were strongly evident in the experiencing people’s narratives and perhaps reflects a directness less present in the UK than China. Interestingly a difference between Fox and Diab’s [7] work and this study is that none of the Chinese participants discussed staff members’ views on their recovery, whereas participants in the UK context reported being attuned to staff members’ pessimism about the likelihood of recovery.

In common with another study from the UK [9], Chinese participants also reported a culture of restriction, which they described as excessive ‘strictness’ and perceived the inpatient unit as an alternate reality, similar to the ‘bubble’ described in Seed et al.’s [12] UK sample. Haynes et al.’s [8] sample and the Chinese participants both reported using relational strategies to cope with the ‘bubble’ of hospitalization, such as being overly compliant and cooperative, secretive behaviors around eating and competing with other experiencing people. This compliance seemed to be associated with a loss of voice and a parallel between the use of ED behaviors which may have been employed to gain a sense of control over oneself and the punitive measures like mechanical restraint, albeit intended to save lives, which seemed to also be an attempt to control the experiencing person.

For some of the Chinese participants, hospitalization offered a means of acquiring new ED behaviors and they used their peers’ knowledge to develop new behaviors, for example, around purging. This unhealthy modelling is an experience reported in other inpatient settings [42, 43]. It could be suggested that difficulties in asserting boundaries between oneself and others’ behaviors might be understood to some degree by discussions around identity and it was interesting to us (both authors) that the Chinese participants did not discuss the impact of treatment on their identity and its development. As authors, we reflected on how this identity during treatment is a common narrative in the UK context (AH, second author), whereas individual identity may be less evident in the Chinese context (YW, first author).

It was interesting to note that a positive outcome for the Chinese participants was feeling more able to eat with others and live a ‘normal’ life (in their own words) and this experience is similar to that of the Dutch adolescents in Van Ommen et al.’s [14] study. Like the Dutch participants who used individual action plans, the Chinese participants reported how helpful the meal plans were to enable them to achieve ‘a normal life’. Like Kezelman et al. [16] the Chinese participants reported strong ambivalence around re-feeding and weight gain and expressed needing more support with the anxiety this caused. In common with Pemberton and Fox’s [11] theme entitled ‘looking for ideal care,’ derived from their interviews with participants in the UK, the master theme reflecting perceptions of treatment received by the Chinese participants also suggests that this participant group may have had an ideal treatment plan in mind. While the master theme in this study reflecting the impact of treatment suggests that physical rehabilitation was achieved, and indeed some participants even felt that their lives had been saved, they were unhappy that their psychological needs had been under-addressed as for them, this was a vital part of ideal care. On the other hand, it was difficult to know whether participants had an idealized view of care, or the degree to which their expectations of ideal care were realistic. Nonetheless, it is important to hear participants’ desire for a tailored treatment focused on both physical and psychological recovery. National Institute for Care Excellence (NICE) guidelines [17] recommend a range of evidence-based treatments for EDs such as CBT [44], Family Based Treatment [45] and the Maudsley Model of Anorexia Treatment for Adults [46] although none of the Chinese participants discussed having received interventions beyond re-feeding and medication. While this reflects an opportunity to share good practice with international colleagues, it should be noted that the evidence base for these treatments and the context for the NICE Guidelines comes largely from UK, USA, and Australian studies and more research is needed on their efficacy in, for example, China.

Taken together, despite some important differences like participants’ reports regarding the use of mechanical restraint and sedation to manage ED behaviors and negligible psychological intervention, which these experiencing individuals experienced as traumatic, the experiences of the Chinese participants in treatment for EDs are rather similar to those of participants studied in other countries (largely the UK, USA, Australia and the Netherlands).

There are some limitations which need to be considered when interpreting these findings. The generalizability of the findings is limited by the inclusion of young females with the binge-purge subtype of AN and further work is needed to explore whether these participants’ of inpatient care are shared by those with other forms of EDs. The study participants were adolescents and developmentally, are highly susceptible to peer influence [47] and social evaluation [48]. Therefore their perceptions relating to these facets might be over-reported or biased. Further work should explore the reliability of these themes across the lifespan. Although the sample size is small, it provides adequate narratives for the idiographic nature of IPA [36]. The translation of the interviews from Chinese into English may have resulted in issues with vocabulary selection. However, there were some benefits noted from conducting the interviews in Chinese. The shared native language of the interviewer and participants reduced participant burden and may have improved the recording of the initial meaning of participants’ narratives. The interviews were conducted at one time-point in the participants’ recovery and future work could explore how their perceptions of inpatient treatment change after a longer period of time. Indeed, further work is needed to explore in more depth the damaging aspects of treatment discussed by participants and how these might impact them in the longer term. As representatives from the medical team at the hospital were not interviewed, it is not possible to know whether participants’ experiences of inpatient treatment corroborate the treatment plan offered. Further work could build on this limitation by interviewing staff and family members to as have Davidson et al., 2019 [49] and Bravender et al., 2017 [50]. This would increase the reliability of the findings and better understand the training needs and anxieties of the staff working with this complex illness. The semi-structured interview tended to confine the construction of participant narratives to questions related to their experiences. Therefore, opportunities to generate data that richly described their identity negotiations was limited in the data collection phases. Future qualitative research is needed to scaffold participant narratives between experience to questions of identity to further analyze some of the effects of inpatient treatments on a person’s identity formation. As our research question was focused on the Chinese patients’ experiences of treatment, the data analysis did not analyse transcript data for the implicit identity negotiations that might be happening in the reported extracts and this would be an important construct to explore in future work with this group. Nonetheless, this work which was the first study to explore the experience of inpatient treatment for an ED in China highlights the usefulness of investigating health issues cross-culturally, as a means of improving global wellbeing based on a deeper understanding of illnesses. It would also be of interest to explore how the experiences of shame and stigma present in some of these experiencing people’s narratives might be understood in these individuals’ cultural context and how this experience might be similar or different to other cultural contexts. Shame, for example, has been discussed by Norwegians experiencing an ED [51].

There are a number of implications arising from this study. The data highlight both similarities and differences between China and other contexts in the treatment of EDs in an inpatient context. Experienced caregivers from the ED field could work to share ideas with international teams around other strategies which can be used to challenge ED behaviors and work collaboratively with those needing/requiring/wanting care. This is particularly important given that data suggest EDs are on the rise in China [28] and collaboration and knowledge-sharing are important tools in fighting global illness. The participants in this study were able to speak with family members through tablet computers, and to further develop these links, the team could be supported to involve families in treatment through information sharing and skills training as this is a low-cost, task sharing approach that has been useful in helping people to remain in the community for longer [52]. Given that the idealization of the thin ideal is also important in China, and this this was salient in the participants’ narratives, international collaborations to influence policy and practice around images on social media, pro-AN websites and ED prevention are warranted. Further exploration of experiences of recovering from an ED which are shared across cultures and/or specific to particular cultures and contexts are needed in the context of the globalized world.

## Conclusions

This qualitative study which is the first study to explore the experiences of inpatient care for an ED in four adolescent females in mainland China found four key themes reflecting both positive and negative experiences of the nature of the treatment received and peer influences, short-term, physical and behavioral improvements and a desire for more psychological treatment, as well as changes to participants’ sense of self, such as perceived stigma associated with medication and mental health service use and improved confidence in the ability to manage ED symptoms in future. Further research into ways of increasing experiencing people’s satisfaction is warranted.

### Reflective section

YW: I have previously fought EDs for several years and suffering such an unforgettable experience intensely motivated me to investigate EDs and to further explore more efficient treatments. Reviewing the research literature, I recognized the impact of inpatient treatment can be controversial. Meanwhile, since I had never received any inpatient treatment, I was extremely interested in understanding inpatient treatment for EDs, particularly regarding what it could actually bring to the participants. I was deeply touched by my participants; they had demonstrated their strong willingness to contribute to the field of study, in order to prevent more people from developing EDs or to evaluate the weaknesses and strengths of inpatient treatment. I was shocked and impressed by the findings. First, the negative effects brought by taking medication significantly outweighed the positive ones, which made me question the most appropriate ways of using medication. Second, the unintentional physical and emotional harm, along with the use of physical restraint, experienced by participants on the ward struck me as extremely inhumane and unethical; though they were people with EDs, they were fundamentally humans deserving of respect. Similar to findings from Western cultures, it seemed hard to reduce or eliminate psychological and cognitive symptoms. What was surprising was that the people receiving treatment on the ward were not always supportive, but teased each other and promoted health-damaging/pro anorexia behaviors. Through regular supervision meetings, I was able to explore my beliefs and be mindful of how they might impact the interpretation of participants’ narratives. It was an extraordinarily valuable experience to conduct this research as a former patient with EDs and a future psychologist.

AH: having worked in the UK in the field of EDs for around 11 years, largely with people with severe and enduring forms of ED on inpatient units, and as a white woman born in the UK with Polish heritage and a clinician researcher, I was really interested to understand how people in another country very different from my own experience of inpatient treatment for EDs. It was interesting to explore the similarities and differences between the UK and Chinese contexts through participants’ testimonies. I was mindful of my western ethnocentric context when conducting the study and analyzing the data, wanting to acknowledge it yet trying to see participants’ experiences through their own eyes. It struck me that there were many similarities between my experiences of being on inpatient units in the UK and the participants in China: participants were ambivalent about change; they desired psychological change yet found it hard to tolerate the physical improvements their bodies needed in order for them to think and feel differently. There were also some differences which for me were distressing: participants reported being mechanically restrained and sedated which made me feel uncomfortable and sad, and through discussions with clinical colleagues I became more mindful of how this was an approach to treatment we have taken in Europe in the past. It was interesting and surprising to hear participants reporting being teased by discharged patients about eating, something I haven’t encountered in the UK. It was a really important learning experience to work with Yi to understand how EDs are understood and treated in her country and this made me reflect more on the diverse patient groups we work with in the UK and how it is vital to be mindful of the ways in which different communities might understand mental illness and treatment.

## Data Availability

The datasets used and/or analyzed during the current study are available from the corresponding author on reasonable request.

## Appendix

### Semi-Structured Interview Guide

1. From your point of view, in what ways has inpatient treatment contributed to your recovery? What was it like being on the ward? What were some of the best and worst things about being in hospital? Was there anything that was particularly helpful or problematic for you?
2. Could you please describe your perception/understanding of the intervention you have received during the period of inpatient care? How has the inpatient admission helped? Please think about this in terms of the bigger picture - your psychological and physiological wellbeing, as well as your quality of life. Could you please provide some examples? The examples could be in general or more specific.
3. How do you perceive the presence of peers who were being hospitalized with you and interacting with you during the period of receiving inpatient care? Any specific examples?
4. Could you discuss the staff you interacted with on the ward during your admission? How did you perceive and experience the support they provided? Can you give any examples to support this?

If you received any medication or other psychological treatment during your admission, how did you experience this? In what ways.

## Abbreviations

AN: Anorexia nervosa
BN: Bulimia nervosa
CBT: Cognitive Behavioral Therapy
EDs: Eating disorders
UK: United Kingdom
